# Novel biomarkers for diagnosis, prognosis, targeted therapy and clinical trials

**DOI:** 10.1186/2050-7771-1-1

**Published:** 2013-01-16

**Authors:** Chung-Tsen Hsueh, Delong Liu, Hong Wang

**Affiliations:** 1 Division of Hematology and Oncology, Loma Linda University, Loma Linda, CA, USA; 2 New York Medical College and Westchester Medical Center, Valhalla, NY 10595, USA; 3 Department of Pharmacology, Temple University School of Medicine, 3420 North Broad Street, Philadelphia, PA 19140, U.S.A

## 

A **biomarker**, as defined by *Wikipedia*, “is an indicator of a biological state. It is a characteristic that is objectively measured and evaluated as an indicator of normal biological processes, pathogenic processes, or pharmacologic responses to a therapeutic intervention.”

## Biomarkers for diagnosis and prognosis

Biomarkers have been used for clinical diagnosis of multiple diseases for many years, for example, troponin level for diagnosing myocardial infarction, creatinine for diagnosing renal insufficiency, and amylase for pancreatitis. Due to the objective values and convenience of measurement with automated technology in the modern era, the applications of biomarkers in biomedicine are increasingly broader and vastly important. Immunophenotyping by flowcytometry study of cell surface markers has become an indispensable tool that hematooncologists and hematopathologists rely on for diagnosing hematological malignancies and monitoring minimal residual diseases. Immunohistostaining of molecular markers have been widely used for identifying cancer types. FLT3 and nucleophosmin (NPM1) mutations are playing important roles in individualizing treatment decisions on acute myeloid leukemia. The applications of gene chips and microarray technology to simultaneously assess multiple molecular markers are catching up to replace single biomarker testing in guiding clinical practice and are being commercialized faster than ever. As an example, Oncotype Dx breast cancer test, a 21-gene panel assay, provides personalized prediction of breast cancer relapse risk and can potentially reduce unnecessary adjuvant chemotherapy.

## Biomarkers for targeted therapy

Targeted agents are revolutionizing the cancer therapy. From monoclonal antibodies (MoAb) to small molecule inhibitors (SMIs) and immunoconjugates, more and more targeted agents are quickly migrating from bench to bedside. Rituximab, a MoAb against CD20, has essentially changed the natural history of diffuse large B cell lymphoma, and is effective in other B-cell disorders such as rheumatoid arthritis. Trastuzumab, a MoAb blocking Her2/neu, is an indispensible agent for Her2/neu positive breast cancer and metastatic gastric cancer. Bevacizumab, a MoAb binding vascular endothelial growth factor (VEGF), has been approved for the treatment of a broad spectrum of advanced malignancies such as colorectal, brain, kidney and non-small cell lung cancer (NSCLC). Brentuximab vedotin (SGN-35), an immunotoxin conjugate, is now available for CD30 positive anaplastic large cell lymphoma and Hodgkin lymphoma. BCR-ABL genetic rearrangement has served as a diagnostic biomarker for chronic myelogenous leukemia (CML). Imatinib, a SMI of ABL, has led the way toward modern-era therapy for CML, essentially changing allogeneic stem cell transplantation to the backseat. The discovery of CD 117 expression on gastrointestinal stromal tumor (GIST) escalated SMI therapy to another level, turning the once most chemo-resistant malignancy to a stunning success of targeted therapy [1,2]. Chemo-resistant renal cell carcinoma (RCC) became sensitive to SMIs, such as sorafenib, sunitinib, and pazopanib. Everolimus and temsirolimus, the class of mTOR inhibitors, have also shown significant activity toward RCC and other malignancies. SMI and MoAb of epidermal growth factor receptor (EGFR) such as gefitinib, erlotinib, cetuximab, panitumumab, are widely used for the treatment of lung, colon, and pancreatic cancers. Crizotinib, an anaplastic lymphoma kinase (ALK) inhibitor, is another biomarker targeting agent against NSCLC harboring ALK translocation, which is mutually exclusive from EGFR mutation.

There are several novel targeted therapies available or in late clinical development for advanced melanoma, one of the most chemo-refractory cancers [3]. Vemurafenib, an inhibitor of BRAF V600E mutant, has been approved for advanced melanoma therapy. Ipilimumab, a MoAb against CTLA-4, has demonstrated efficacy in patients with advanced melanoma. Trametinib, a SMI against MEK, has shown promising activity against BRAF mutated melanoma and may provide an alternative for patients who are resistant to BRAF targeting therapy in the future

JAK2 V617F mutation is the most important biomarker in myeloproliferative neoplasms (MPN). Ruxolitinib, a JAK inhibitor, has been shown to decrease splenomegaly and alleviate symptoms associated with MPN associated with advanced myelofibrosis. More JAK2 –specific inhibitors are undergoing clinical trials.

With the vast knowledge of signal transduction pathways, SMIs are being developed for many known biomarkers, such as PI3K, Bruton’s tyrosine kinase, c-MET, RET, etc.

## Biomarkers as surrogate endpoint for clinical trials

It often takes years to reach the traditional endpoints in clinical trials, such as overall survival, progression free survival, etc. in non-selected patient populations. This is costly and time-consuming. Biomarkers are getting recognitions to serve as surrogate endpoints in cancer trials and help to timely identify patients who will benefit from the specific targeted therapy agents. As an example, BCR-ABL transcript level was recognized as a surrogate biomarker endpoint (major molecular response at 12 months) in ENESTnd trial [4], since it was shown to have correlation with 5-year survival in IRIS trial. It is foreseeable that more biomarkers will replace those time-consuming traditional endpoints, accelerating the translation of novel agents from bench to bedside. Another example is the unprecedented speedy development of vemurafenib for patients with BRAF V600E mutation. It took less than six years from drug discovery to health authority approval for clinical use. Patients with BRAF V600E showed early and dramatic responses to vemurafenib even in a phase I study. Subsequently all phase II and III studies were conducted in selected melanoma patients with BRAF V600E mutation, which confirmed the phase I study findings and demonstrated improved response rate and overall survival in vemurafenib treated patients [5]. Clearly more specific biomarkers will be implemented in the future clinical studies and will help to tailor patient’s best care in a new era of personalized medicine.

To facilitate the growth of this rapidly developing multidisciplinary field, *Biomarker Research* aims to publish original discovery, novel concepts, commentaries, new methodologies, and reviews related to biomarker investigation from all biomedical disciplines.
